# Neuroanatomical Reflections of Childhood Obesity: Volumetric Analysis of the Pituitary Gland and Olfactory Bulb

**DOI:** 10.3390/children12081009

**Published:** 2025-07-31

**Authors:** Emel Hatun Aytaç Kaplan, Elif Bulut, Nazlı Gülsüm Akyel, Zümrüt Kocabey Sütçü, Şeyda Doğantan

**Affiliations:** 1 Department of Pediatric Endocrinology, Başakşehir Çam and Sakura City Hospital, 34480 Istanbul, Turkey; zksutcu@gmail.com; 2 Department of Radiology, Başakşehir Çam and Sakura City Hospital, 34480 Istanbul, Turkey; elifbulut96@gmail.com; 3 Department of Pediatric Radiology, Başakşehir Çam and Sakura City Hospital, 34480 Istanbul, Turkey; nazligulsumakyel@gmail.com; 4 Department of Pediatric Rheumatology, Başakşehir Çam and Sakura City Hospital, 34480 Istanbul, Turkey; drseydacayan@gmail.com

**Keywords:** childhood obesity, magnetic resonance, olfactory bulb, pituitary gland

## Abstract

**Highlights:**

**What are the main findings?**

- Childhood obesity is known to be associated with various metabolic and neuroendocrine alterations.
- Previous studies have shown that obesity affects brain structures such as the olfactory bulb and pituitary gland in adults, but there is limited research on how obesity influences brain volumes in children.

**What is the implication of the main finding?**

- This study provides new insights into volumetric changes in the olfactory bulb and pituitary gland in children with obesity.
- The findings suggest that childhood obesity leads not only to metabolic but also to neuroanatomical changes, potentially reflecting neuroplasticity and endocrine adaptations, highlighting the importance of considering both age and body mass index when evaluating these brain regions.

**Abstract:**

**Introduction:** Obesity is a rapidly increasing condition that leads to serious health issues. The sense of smell, one of the oldest senses related to energy metabolism, has been increasingly studied in relation to obesity. **Objective:** This study investigates the impact of childhood obesity on the volumes of the olfactory bulb and pituitary gland, exploring the relationship between body mass index and these brain structures. **Method:** This study included 146 participants aged 6–18 years with different body mass indices between 2021 and 2024 at Basaksehir Cam and Sakura City Hospital, Istanbul, Turkey. Participants were classified into normal weight, obese, and morbidly obese groups, and olfactory bulb and pituitary gland volumes were retrospectively analyzed. MRI scans were performed to exclude intracranial pathologies due to headache complaints, and patients with cranial pathologies were excluded from the study. **Results:** This study examined the olfactory bulb and pituitary gland volumes among normal weight, obese, and morbidly obese groups aged 6–18 years. In the morbidly obese group, right olfactory bulb area and right olfactory bulb volume were significantly higher compared to the other groups, while left olfactory bulb area was higher in both the obese and morbidly obese groups. Additionally, in the morbidly obese group, pituitary height was significantly lower than the other groups, and pituitary volume was also found to be reduced in morbid obesity. **Conclusions:** This study demonstrated that childhood obesity is linked to significant changes in the volumes of the olfactory bulb and pituitary gland. In morbidly obese children, an increase in pituitary volume and alterations in olfactory bulb volume suggest possible neuroanatomical adaptations.

## 1. Introduction

Obesity is a rapidly increasing condition that leads to serious health issues. Obesity is a significant condition with increasing prevalence in recent years and is a major cause of morbidity and mortality [1]. One of the key contributors to this rise is the easy accessibility of high-calorie foods and the widespread promotion and preference for palatable, energy-dense meals [2]. The sense of smell is one of the most evolutionarily conserved and ancient senses in living organisms [3]. There is a strong relationship between olfactory function and the regulation of energy metabolism in the body [4]. It has been suggested that olfactory sensitivity may be linked to adaptation to high-calorie dietary intake [5]. The olfactory bulb, known as the central structure of olfaction, also plays roles in energy regulation and memory processing beyond its primary function. The volume of the olfactory bulb can vary depending on several factors [6]. Previous animal studies have examined the association between genomic alterations in the brain and obesity. A possible relationship has been proposed between obesity and genomic changes in the olfactory bulb and striatum [7]. Most of these studies focus on cellular- and neural-level changes [8]. Although there are studies investigating the relationship between olfactory perception and obesity, they are limited in their explanations of the underlying mechanisms. Particularly, studies directly examining the association between olfactory bulb volume (OBV) and obesity are scarce.

The hypothalamus is the key region responsible for regulating hunger and satiety. Lesions in areas such as the ventromedial hypothalamus, paraventricular hypothalamus, lateral hypothalamus, or arcuate nucleus (due to inflammation, trauma, tumors, etc.) may result in hyperphagia, decreased basal metabolic rate, sleep disturbances, and obesity [9]. Recent advancements in magnetic resonance imaging (MRI) technology have greatly contributed to the diagnostic evaluation of various neurological, oncological, and endocrine disorders [10]. It is well-established that pituitary gland size and morphology are associated with endocrine disorders, particularly in cases of growth retardation [10,11]. Pituitary volume analyses have also been conducted in conditions such as depression, schizophrenia, polycystic ovary syndrome, substance abuse, and hormone deficiencies [12,13,14,15]. Since pathologies in the hypothalamic–pituitary axis can lead to hormonal imbalances, a potential link with obesity is plausible. There are normative pituitary measurements established based on age and sex [14]. However, studies investigating the relationship between obesity and pituitary gland height remain limited.

In this study, we aimed to explore the relationship between the olfactory bulb, pituitary gland, and obesity. Specifically, we focused on childhood obesity, an increasingly prevalent public health issue, and aimed to assess the associations between body mass index (BMI) and pituitary gland height, pituitary volume, and olfactory bulb volume.

## 2. Materials and Methods

A total of 146 participants aged between 6 and 18 years, with varying body mass indices, were included in our study between the years 2021 and 2024. Approval was obtained from the local ethics committee of our hospital (protocol code: KAEK/17.01.2024.31). Written informed consent was obtained from the legal guardians of all participants prior to MRI scanning. Participants were categorized into three groups: normal weight, obese, and morbidly obese. The classification was based on weight-for-height percentiles and body mass index (BMI). Height and weight measurements were performed by experienced healthcare professionals. BMI, weight percentiles, and anthropometric measurement Z-scores were calculated according to age and sex using the Child Metrics software (version 1.0.1).

Olfactory bulb volumes (OBVs) and pituitary measurements of the groups were evaluated retrospectively. MRI scans were performed to rule out intracranial pathology in patients presenting with headache. Patients with cranial pathologies were excluded from the study. The MRI images were assessed blindly and independently by two experienced radiologists, unaware of patient clinical data. The evaluations included left and right olfactory bulb volumes, pituitary height, and pituitary volume.

Exclusion criteria were as follows: presence of brain parenchymal pathology, malignancy, history of trauma, neuropsychiatric disorders, anosmia/hyposmia, chronic systemic diseases, diagnosed migraine, steroid use, diagnosis of diabetes mellitus, and history of cerebrovascular events.

### 2.1. MRI Method

A total of 146 participants were scanned using a 3T MR machine (Ingenia; Philips Medical Systems; Best, The Netherlands). The routine brain MRI protocol included sagittal 3D T1-weighted (T1W) turbo spin echo (TSE) (repetition time (TR): 25 milliseconds (ms), echo time (TE): 3.9 ms, slice thickness (st): 1.1 mm, field of view (FOV): 200 mm, matrix: 200 × 200 mm, gap: 0), axial T2-weighted (T2W) TSE (TR: 6192 ms, TE: 110 ms, st: 5 mm, FOV: 200 mm, matrix: 236 × 135 mm, gap: 1), coronal T2W TSE (TR: 7619 ms, TE: 110 ms, st: 4.5 mm, FOV: 180 mm, matrix: 200 × 166 mm, gap: 1), axial 3D FLAIR (TR: 4800 ms, TE: 339 mm, st: 3 mm, FOV: 200 mm, matrix: 200 × 164 mm, gap: 0), axial susceptibility-weighted imaging (SWI) (TR: 15 ms, TE: 21 ms, st: 2 mm, FOV: 197 mm, matrix: 196 × 160 mm, gap: −1), diffusion-weighted imaging (DWI) (TR > 4030 ms, TE > 76 7.5 ms, st: 4 mm, FOV: 156 mm, matrix: 100 × 97 ms, gap: 1), and apparent diffusion coefficient (ADC) maps.

All MRI images were retrospectively evaluated. The examinations were reviewed by radiologists (E.B., N.G.A.). Pituitary volumes were measured by E.B., and olfactory bulb volumes by N.G.A., ensuring consistency in evaluation. The height and width of the pituitary gland were measured at the midline on the coronal T2W sequence. The anteroposterior (AP) diameter was measured on the sagittal T1W sequence to avoid including the neurohypophysis. Pituitary volume was calculated using the ellipsoid formula: Volume = (height × width × AP diameter)/2. All volume measurements are expressed in mm^3^.

**Figure 1:**
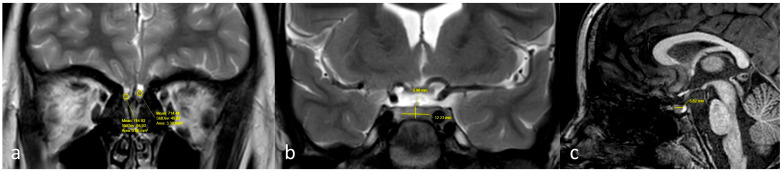
Measurement of olfactory bulbs and pituitary gland. (**a**) Olfactory bulb areas were measured on coronal T2W images by manual tracing. (**b**) Pituitary gland height and width were measured on coronal T2W images. (**c**) The AP diameter was measured on sagittal 3D T1W images. The participant shown in this example was an 8-year-old girl with morbid obesity.

Olfactory bulb volume was calculated using a stereological method. The olfactory bulb area was measured on the coronal T2W sequence by tracing in the PACS system. As the olfactory bulb typically appeared on a single slice due to its small size, the volume of the bulb was calculated using the following formula: Volume = calculated area (mm^2^) × slice thickness (4.5 mm). Figure 1 shows the volumetric measurements of the olfactory bulbs and the pituitary gland (Figure 1).

### 2.2. Statistical Analysis

Study data were analyzed using SPSS version 25.0 (IBM Corp., Armonk, NY, USA). Descriptive statistics were presented as mean ± standard deviation (SD) for continuous variables and as frequency and percentage (%) for categorical variables. Categorical variables were compared between groups using the Chi-square test. Pearson correlation analysis was conducted based on the distribution of the variables.

Normality was assessed via the Shapiro–Wilk test. Normally distributed data were presented as mean ± SD with 95% CI, while non-normal data were shown as median (IQR) with min–max values. Box plots displayed medians (horizontal line), quartiles (box boundaries), and ranges (whiskers). Group comparisons used ANOVA with Tukey HSD for normal data and Kruskal–Wallis with Mann–Whitney U tests for non-normal data. Pearson or Spearman correlations were calculated based on distribution. Bonferroni correction was applied for multiple comparisons. Statistical significance was set at *p* < 0.05.

## 3. Results

The mean age of the study groups was as follows: all participants: 13.95 ± 2.47 (median = 14.1 IQR: 7.09–17.91) (66.4% girls). The normal weight group (n = 67; 42 girls, 25 boys) had a mean age of 14.18 ± 2.36 years, while the obese and morbidly obese group (n = 79; 55 girls, 24 boys) had a mean age of 13.59 ± 2.57 years. Age and sex distributions were similar between the groups. The height and weight values of all participants are presented in the table (Table 1). The weights and body mass indices (BMIs) of the study group were significantly higher than those of the control group.

When participants were grouped as normal weight, obese, and morbidly obese, the right olfactory bulb area (ROBA) was found to be significantly higher in the morbidly obese group compared to the other groups. The left olfactory bulb area (LOBA) was significantly higher in both the obese and morbidly obese groups compared to the normal weight group (*p* < 0.01). According to the Mann–Whitney U test results, the ROBA values of the morbidly obese group were significantly different from those of the other two groups (*p* < 0.05). Post hoc analyses for the LOBA variable revealed a statistically significant difference between the normal weight group and both the obese (*p* < 0.05) and morbidly obese (*p* < 0.001) groups.

**Figure 2:**
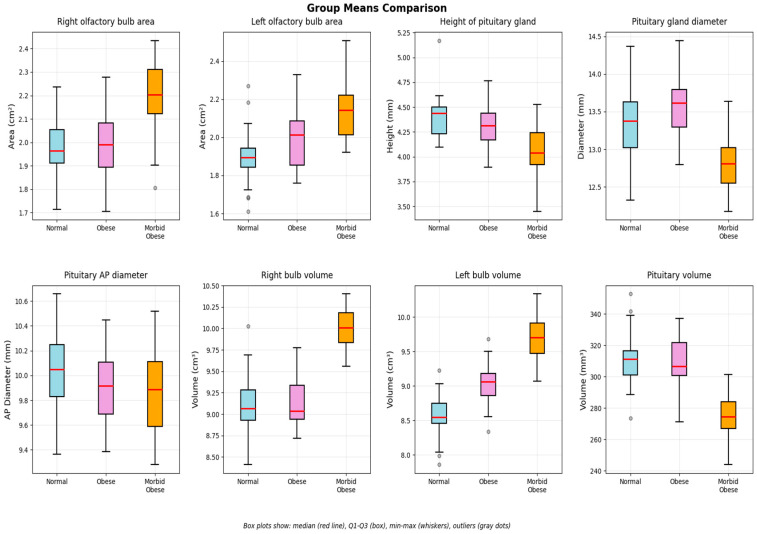
Box plots showing the distribution of significant variables across the three groups. The boxes represent the interquartile range, with the median line inside. Whiskers extend to the most extreme data points, and outliers are shown as individual points.

**Figure 3:**
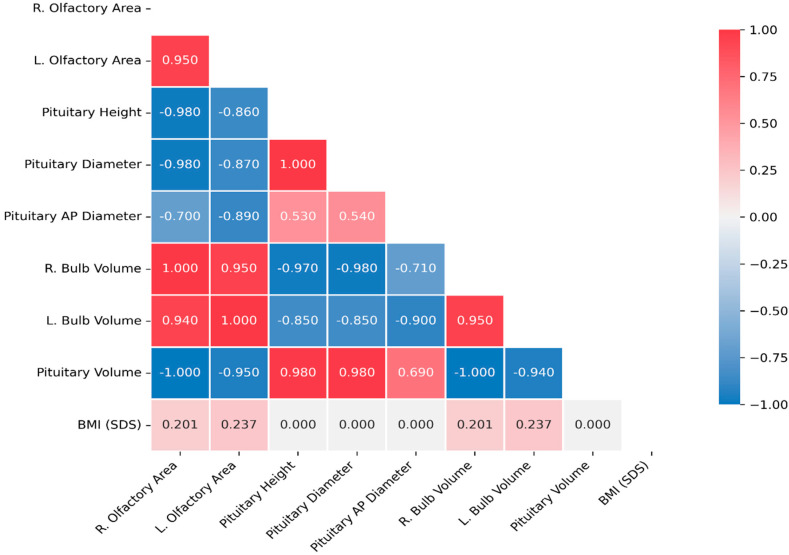
Correlation heatmap showing relationships between all measured variables. Red colors indicate positive correlations; blue colors indicate negative correlations. The correlation coefficients are displayed in each cell. R: right; L: left.

The right olfactory bulb volume (ROBV) was similar in the normal weight and obese groups, but significantly higher in the morbidly obese group compared to both. The Tukey HSD post hoc test showed that this difference in ROBV was statistically significant only between the normal weight and morbidly obese groups (*p* < 0.05). The left olfactory bulb volume (LOBV) was also similar between the normal and obese groups, but significantly higher in the morbidly obese group compared to the normal weight group (*p* < 0.01). The height of the pituitary gland (craniocaudal diameter) was similar between the normal weight and obese groups but was significantly lower in the morbidly obese group. The mediolateral and anteroposterior diameters of the pituitary gland were comparable across all three groups. Pituitary volume was significantly lower in the morbidly obese group than in the normal weight and obese groups. The values of the variables across the groups are presented in Table 2 and illustrated in Figure 2 and Figure 3. In our study, significant correlations were found between body mass index (BMI) and both right and left olfactory bulb volumes. Additionally, a strong correlation was observed between the right and left olfactory bulb volumes (Table 3).

## 4. Discussion

In this study, we investigated the volumetric changes in the olfactory bulb and pituitary gland in children with obesity. In this study, olfactory bulb volumes were larger in obese children compared to normal weight children, while in the morbidly obese group, these volumes unexpectedly increased. A strong correlation was found between BMI and olfactory bulb volumes. Additionally, pituitary volume was significantly reduced in the morbidly obese group. These findings suggest that obesity-related hormonal changes and chronic inflammation may lead to structural changes in the olfactory bulb and pituitary gland.

The olfactory sense, known as the oldest sensory reflex in evolutionary history, has been well-preserved from primitive times to the present day [16]. Olfactory neurons possess a unique structure that facilitates the chemical exchange of information between the brain and the external environment [17]. This system emerges during the intrauterine period and is a well-preserved structure with its own specialized ganglion throughout life [17]. The olfactory system is active in processes such as adolescence, memory, cognitive functions, and reproduction [6,18].

Changes in the size of an organ may lead to changes in the organism, either directly or indirectly, and vice versa. This concept can provide insight into how energy allocation is directed according to the body’s needs. Unused organs may shrink, while organs that are highly utilized may experience growth [19]. The plasticity of the olfactory bulb is also related to the amount of stimulation received by the olfactory receptors. Environmental stimuli and olfactory sensitivity can alter the olfactory bulb volume [20]. The increased olfactory bulb volume observed in individuals with obesity may be related to neuronal plasticity induced by alterations in metabolic hormones such as leptin and insulin. Additionally, chronic low-grade neuroinflammation has been suggested to contribute to structural changes in the olfactory system. This study showed that there could be changes in the olfactory bulb and pituitary size in obesity.

In adult patients who underwent laryngectomy, OBV, which was initially reduced, showed an increase in size over time with improved sensitivity following olfactory rehabilitation. This finding supports the plasticity of the olfactory bulb [21]. In a study by Poessel et al., OBV was significantly lower in obese participants compared to those with a normal weight, and this was associated with a gradual decline in olfactory sensitivity with weight gain [22]. Studies in adults support the idea that OBV decreases with excessive weight gain, and the lack of support for this trend in our study may be due to the fact that our participants were children, who may not have had enough time for the olfactory bulb to shrink.

It is known that lateralization occurs in the olfactory development process, with the right olfactory bulb being more dominant than the left [23]. In our study, however, no significant lateralization was found. Both sides of the olfactory bulb were significantly larger in the morbidly obese group. Interestingly, the left olfactory bulb area (LOBA) was larger not only in the morbidly obese group but also in the obese group. Similarly, Karaoğlan et al. [24] did not support the concept of lateralization in their study, which aligns with our findings. An important observation in our study was that while the olfactory bulb volume increased in the obese group, it was unexpectedly reduced in the morbidly obese group. This was explained by the authors as being a result of reduced olfactory sensitivity in morbidly obese individuals [23]. However, in our study, all olfactory bulb measurements were higher in the morbidly obese group compared to the other groups. We cannot explain this finding through the olfactory sensitivity hypothesis. In our study, significant correlations were found between BMI and both right and left olfactory bulb volumes. Additionally, a strong correlation was observed between the right and left olfactory bulb volumes.

The hypothalamus–pituitary–adrenal axis is intricately linked to obesity. A recent study demonstrated a significant connection between BMI and the pituitary gland’s functional MRI in key brain regions [25]. Our study addressed the pituitary morphology observed in routine clinical MRI scans. Limited studies discussing the relationship between pituitary volume and obesity have yielded contradictory results. In one adult study, pituitary volume was found to increase in morbid obesity [26]. In a study involving childhood patients, pituitary height was found to be higher in severe obesity; however, no correlation was found between the BMI Z-score and pituitary height [24]. In another study, pituitary volume was similar in normal weight and obese participants initially, but after three years of follow-up, the pituitary volume significantly decreased in those who remained obese. This study emphasized the role of weight gain in reducing pituitary volume. A three-year prospective investigation proposed the hypothesis that pituitary modifications could be a result rather than a cause of obesity [27]. In our study, pituitary volume and height were similar in normal weight and obese children, whereas they were significantly lower in the morbidly obese group. There were no significant differences in other pituitary measurements between the groups. Our findings strongly support the hypothesis by Puliani et al. suggesting that endocrinopathies in morbid obesity may be explained by this hypothesis. In this population, the brain is still undergoing active development, and obesity-related factors—such as inflammation, insulin resistance, and hormonal alterations—may interfere with or accelerate certain neurodevelopmental processes. These effects could contribute to increased volumes in specific brain regions. However, due to inconsistencies and contradictions in the existing literature, our ability to comprehensively interpret these findings remains limited. While it is undeniable that changes in the hypothalamus–pituitary axis could occur in obesity, this information still needs further support and cellular-level investigation.

For longitudinal studies, we recommend tracking changes in brain structure and function over time in morbidly obese children and adolescents. Such studies would help to better understand how obesity-related factors like inflammation, metabolic disturbances, and hormonal changes affect brain development at different growth stages. Additionally, including BMI measurements at multiple time points could provide insight into the critical periods when these changes are most pronounced.

Regarding functional studies, investigating how obesity influences brain activity and cognitive function using neuroimaging techniques like functional MRI (fMRI) or electrophysiological methods would be highly valuable. These studies could identify brain regions associated with cognitive impairments or behavioral changes in obese individuals. Combining structural and functional neuroimaging data would allow for a more comprehensive understanding of the long-term effects of obesity on the brain.

We believe that both longitudinal and functional studies will contribute to the design of targeted interventions for children and adolescents with obesity, helping to mitigate potential long-term neurological consequences.

### Limitations

This study’s cross-sectional design limits the ability to establish causal relationships. The inclusion of only pediatric participants restricts the evaluation of age-related structural changes. Imaging was based on routine clinical MRI, without advanced volumetric analysis. Additionally, there was no functional assessment of the olfactory or pituitary systems, and no long-term follow-up data were available.

## 5. Conclusions

This study showed that childhood obesity is associated with significant structural changes in the volumes of the olfactory bulb and pituitary gland. Specifically, a decrease in pituitary volume was observed in morbidly obese children, and alterations in olfactory bulb volume may have reflected underlying plasticity, indicating the importance of considering age, sex, and body mass index when evaluating these neuroanatomical structures.

## Figures and Tables

**Table 1:** Anthropometric measurements of the participants. Statistical summary of anthropometric variables with distribution analysis.

		N	Mean	Std Distribution	95%CI	Deviation	Test	*p*-Value
Height (cm)	Normal Weight	67	159.78	11.67	156.99–162.57	Normal	ANOVA	0.54
	Obesity	40	159.78	9.35	156.88–162.68	Normal		
	Morbid Obesity	39	157.36	14.13	152.93–161.79	Normal		
Height SDS	Normal Weight	67	0.03	1.15	−2.27–2.33	Non-normal	K-W	0.12
	Obesity	40	0.19	1.05	−0.14–0.52	Normal		
	Morbid Obesity	39	0.49	1.36	0.06–0.92	Normal		
Weight (kg)	Normal Weight	67	54.2	13.15	51.05–57.35	Normal	ANOVA	* 0.00
	Obesity	40	66.8	10.67	63.49–70.11	Normal		
	Morbid Obesity	39	82.2	24.17	74.61–89.79	Normal		
Weight SDS	Normal Weight	67	0.05	1.18	−0.23–0.33	Normal	ANOVA	* 0.00
	Obesity	40	1.67	0.76	1.43–1.91	Normal		
	Morbid Obesity	39	3.18	1.02	2.86–3.50	Normal		
BMI percentile	Normal Weight	67	52.7	26.53	46.35–59.05	Normal	ANOVA	* 0.00
	Obesity	40	94.58	3.5	93.50–95.66	Normal		
	Morbid Obesity	39	99.44	0.74	99.21–99.67	Normal		
BMI SDS (kg/m^2^)	Normal Weight	67	0.1	0.84	−0.10–0.30	Normal	ANOVA	* 0.00
	Obesity	40	1.7	0.36	1.59–1.81	Normal		
	Morbid Obesity	39	2.87	0.6	2.68–3.06	Normal		
Weight percentage according to height (%)	Normal Weight	67	103.44	9.01	101.28–105.60	Normal	ANOVA	* 0.00
	Obesity	40	128.59	5.69	126.83–130.35	Normal		
	Morbid Obesity	39	163.35	21.51	156.60–170.10	Normal		

Independent Samples Test. SDS: standard deviation score, BMI: body mass index. Values are presented as mean ± SD for normally distributed variables and median (IQR) for non-normally distributed variables; 95% CI: 95% confidence interval for means or min–max range for medians. K-W: Kruskal–Wallis test; ANOVA: Analysis of Variance. * *p* < 0.001.

**Table 2:** Comparison of olfactory bulb and pituitary gland measurements among study groups.

Variable	Group	n	Value	95% CI	Distribution	Test	*p*-Value
ROBA (mm^2^)	Normal Weight	67	2.02 ± 0.53	[1.89–2.15]	Normal	ANOVA	0.046 *
	Obesity	40	2.04 ± 0.54	[1.87–2.21]	Normal		
	Morbid Obesity	39	2.25 ± 0.62	[2.06–2.44]	Normal		
LOBA (mm^2^)	Normal Weight	67	1.91 (1.54–2.28)	[0.81–3.01]	Non-normal	K-W	0.031 *
	Obesity	40	2.00 ± 0.48	[1.85–2.15]	Normal		
	Morbid Obesity	39	2.14 ± 0.51	[1.98–2.30]	Normal		
ROBV (mm^3^)	Normal Weight	67	9.08 ± 2.40	[8.51–9.65]	Normal	ANOVA	0.015 *
	Obesity	40	9.19 ± 2.44	[8.43–9.95]	Normal		
	Morbid Obesity	39	10.14 ± 2.78	[9.27–11.01]	Normal		
LOBV (mm^3^)	Normal Weight	67	8.58 ± 2.49	[7.98–9.18]	Normal	ANOVA	0.040 *
	Obesity	40	9.01 ± 2.17	[8.34–9.68]	Normal		
	Morbid Obesity	39	9.61 ± 2.29	[8.89–10.33]	Normal		
Height—PG (mm)	Normal Weight	67	4.40 ± 1.18	[4.12–4.68]	Normal	ANOVA	0.015 *
	Obesity	40	4.45 ± 1.31	[4.04–4.86]	Normal		
	Morbid Obesity	39	4.06 ± 1.03	[3.74–4.38]	Normal		
Pituitary ML diameter (mm)	Normal Weight	67	13.31 ± 2.16	[12.79–13.83]	Normal	ANOVA	0.040 *
	Obesity	40	13.36 ± 2.18	[12.68–14.04]	Normal		
	Morbid Obesity	39	12.94 ± 1.99	[12.32–13.56]	Normal		
Pituitary AP diameter (mm)	Normal Weight	67	10.01 ± 1.12	[9.74–10.28]	Normal	ANOVA	0.200
	Obesity	40	9.94 ± 1.32	[9.53–10.35]	Normal		
	Morbid Obesity	39	9.93 ± 1.42	[9.48–10.38]	Normal		
Pituitary volume (mm^3^)	Normal Weight	67	314.12 ± 124.40	[284.33–343.91]	Normal	K-W	0.040 *
	Obesity	40	311.02 ± 117.28	[274.67–347.37]	Normal		
	Morbid Obesity	39	275.07 (208.88–341.26)	[78.95–471.19]	Non-normal		

ROBA: right olfactory bulb area, LOBA: left olfactory bulb area, ROBV: right olfactory bulb volume, LOBV: left olfactory bulb volume, PG: pituitary gland, ML: mediolateral, AP: anteroposterior. Values are presented as mean ± SD for normally distributed variables and median (IQR) for non-normally distributed variables; 95% CI: 95% confidence interval for means or min–max range for medians. K-W: Kruskal–Wallis test; ANOVA: Analysis of Variance. * *p* < 0.05.

**Table 3:** Correlations between body mass index (BMI) and participants’ pituitary gland and olfactory bulb volumes.

		ROBA (mm^2^)	LOBA (mm^2^)	Height—PG (mm)	Pituitary ML Diameter (mm)	Pituitary AP Diameter (mm)	ROBV (mm^3^)	LOBV (mm^3^)	Pituitary Volume (mm^3^)	BMI
ROBA (mm^2^)	Pearson Correlation	1	0.861 **	0.009	0.147	0.064	1000 **	0.861 **	0.098	0.201 *
	Sig. (2-tailed)		0.000	0.911	0.078	0.444	0.000	0.000	0.237	0.015
	N	146	146	146	146	146	146	146	146	146
LOBA (mm^2^)	Pearson Correlation	0.861 **	1	0.011	0.130	0.001	0.861 **	1000 **	0.068	0.237 **
	Sig. (2-tailed)	0.000		0.898	0.119	0.992	0.000	0.000	0.414	0.004
	N	146	146	146	146	146	146	146	146	146
Height—PG (mm)	Pearson Correlation	0.009	0.011	1	0.219 **	−0.002	0.009	0.011	0.802 **	−0.039
	Sig. (2-tailed)	0.911	0.898		0.008	0.986	0.911	0.898	0.000	0.644
	N	146	146	146	146	146	146	146	146	146
Pituitary ML diameter (mm)	Pearson Correlation	0.147	0.130	0.219 **	1	0.322 **	0.147	0.130	0.658 **	−0.026
	Sig. (2-tailed)	0.078	0.119	0.008		0.000	0.078	0.119	0.000	0.755
	N	146	146	146	146	146	146	146	146	146
Pituitary AP diameter (mm)	Pearson Correlation	0.064	0.001	−0.002	0.322 **	1	0.064	0.001	0.433 **	−0.080
	Sig. (2-tailed)	0.444	0.992	0.986	0.000		0.444	0.992	0.000	0.340
	N	146	146	146	146	146	146	146	146	146
ROBV (mm^3^)	Pearson Correlation	1000 **	0.861 **	0.009	0.147	0.064	1	0.861 **	0.098	0.201 *
	Sig. (2-tailed)	0.000	0.000	0.911	0.078	0.444		0.000	0.237	0.015
	N	146	146	146	146	146	146	146	146	146
LOBV (mm^3^)	Pearson Correlation	0.861 **	1000 **	0.011	0.130	0.001	0.861 **	1	0.068	0.237 **
	Sig. (2-tailed)	0.000	0.000	0.898	0.119	0.992	0.000		0.414	0.004
	N	146	146	146	146	146	146	146	146	146
Pituitary Volume (mm^3^)	Pearson Correlation	0.098	0.068	0.802 **	0.658 **	0.433 **	0.098	0.068	1	−0.096
	Sig. (2-tailed)	0.237	0.414	0.000	0.000	0.000	0.237	0.414		0.250
	N	146	146	146	146	146	146	146	146	146
BMI	Pearson Correlation	0.201 *	0.237 **	−0.039	−0.026	−0.080	0.201 *	0.237 **	−0.096	1
	Sig. (2-tailed)	0.015	0.004	0.644	0.755	0.340	0.015	0.004	0.250	
	N	146	146	146	146	146	146	146	146	146

BMI: body mass index, ROBA: right olfactory bulb area, LOBA: left olfactory bulb area, ROBV: right olfactory bulb volume, LOBV: left olfactory bulb volume, PG: pituitary gland, ML: mediolateral, AP: anteroposterior. **: Correlation is significant at the 0.01 level (2-tailed). *: Correlation is significant at the 0.05 level (2-tailed).

## Data Availability

The original contributions presented in this study are included in the article. Further inquiries can be directed to the corresponding author.
